# Characteristics of the pseudorabies virus strain GDWS2 with severe neurological signs and high viral shedding capacity in pigs

**DOI:** 10.3389/fvets.2025.1530765

**Published:** 2025-04-14

**Authors:** Wang Chen, Gao Fan, Yurong Huang, Keyue Zhou, Zifan Chen, Kexin Chen, Huihua Zhang, Zhaoyao Li

**Affiliations:** ^1^School of Animal Science and Technology, Foshan University, Foshan, China; ^2^Wen’s Food Group, Yunfu, China; ^3^College of Veterinary Medicine, South China Agricultural University, Guangzhou, China

**Keywords:** pseudorabies virus, GDWS2 strain, neurological signs, respiratory symptoms, phylogenetic analysis, viral recombination, pig industry

## Abstract

Pseudorabies virus (PRV) poses a serious threat to the global swine industry, as PRV infection can lead to reproductive disorders in sows and high mortality in newborn piglets. Although pigs typically exhibit age-related resistance to PRV, with older pigs exhibiting milder symptoms upon infection, the recent isolation of multiple highly pathogenic PRV variants and reports of severe symptoms and even death in older pigs have garnered much attention. The GDWS2 strain isolated in this study exhibits characteristics similar to those of highly pathogenic strains. GDWS2 was isolated from the brain tissue of a 90-day-old diseased pig that exhibited severe respiratory and neurological symptoms. The pig originated from a farm that had been previously vaccinated with the Bartha-k61 strain. *In vitro* experiments demonstrated that GDWS2 induces substantial cytopathic effects in PK-15, VERO, BHK cells, and PAM. Moreover, GDWS2 formed larger plaques and exhibited higher early replication titers in PK-15 cells compared to the highly pathogenic variant strain JM isolated in China. Phylogenetic analysis revealed that GDWS2 belongs to PRV genotype II, with *gD*, *gE*, and *TK* genes showing high homology to those of highly pathogenic PRV variants. Additionally, GDWS2 harbors unique insertions or mutations in the *US1*, *UL36*, and *UL5* gene regions, and its genome contains recombination events with PRV variants, Bartha, or genotype I strains. *In vivo* experiments further confirmed the high pathogenicity of GDWS2. In rabbit and 90-day-old pig models, GDWS2, compared with the JM strain, caused high mortality rates, accompanied by severe pathological damage. Notably, in the 90-day-old pig model, the GDWS2 challenge group exhibited more severe respiratory and neurological symptoms, and enhanced neurotropism and shedding capacity. The data from this study may indicate the emergence of a naturally recombined and highly pathogenic PRV variant in China once again.

## 1 Introduction

Pseudorabies virus (PRV), also known as porcine herpesvirus type I, belongs to the subfamily Herpesviridae and the genus Var*icellovirus*. It is one of the major swine-farming infectious diseases, causing great losses to the global animal husbandry industry (1). It is an enveloped, double-stranded DNA virus with a genome size of approximately 140 kbp (2). The genome encodes proteins essential for the virus capsid, tegument, and envelope, including glycoproteins responsible for virulence (gB, gC, gD, gE, gL, gG, gM, gN, and TK) (3, 4). In recent years, it has been reported that nonvirulence genes such as *US1*, *UL36*, and *UL5* play crucial roles in virus replication (5), assembly process, neural invasion (6), early infection of host cells, and evasion of the host’s immune defense (7). The *gC* gene is usually used to distinguish between genotype I and genotype II. In China, genotype II exists widely as the predominantly prevalent strain (8).

The first case of PRV infection in China can be traced back to a domestic cat in 1947 (9). Due to the lack of effective PRV vaccines and biosafety prevention and control measures at that time, the virus spread rapidly among pig populations in several regions of China. The Bartha-k61 strain, an attenuated strain obtained through cell passage attenuation technology, demonstrated good immunoprotective effects against the prevalent classic PRV strains (e.g., Ea, Fa, and SC strains) after it was introduced in the 1970s in China (10–12). However, since 2011, PRV outbreaks have occurred in multiple pig farms that had been vaccinated with the Bartha-k61 vaccine (13). Ever since, PRV variant strains (e.g., JS-2012, HB1201 and HeN1) have gradually become the dominant strains in China, causing huge economic losses to the pig-farming industry (8, 14–16). Multiple studies have demonstrated that the protective effect of the Bartha-k61 vaccine against PRV variants (e.g., TJ, ZJ01, SMX) is limited (16–18). At present, several candidate vaccines developed against PRV variant strains have demonstrated significant protective efficacy in mouse and pig models. These vaccines primarily include gene-deleted vaccines (16, 19, 20), subunit vaccines (21, 22), DNA vaccines (23, 24), and mRNA vaccines (25). However, the global epidemiological landscape of PRV remains highly complex. Reports indicate that between 2011 and 2021, the overall positive rate of PRV wild strains in China reached 29.87% (76,553/256,326) (26). Additionally, Argentina and France experienced secondary PRV outbreaks in 2019 and 2020, respectively.^1^ In recent years, the host range of animal-origin PRV infections has been increasing (27). An outbreak in Italy’s Campania region continued to expand, with reports of hunting dogs exhibiting symptoms such as fever, self-inflicted head wounds, and ataxia following contact with wild boars (27). This highlights the risk of PRV transmission between domestic and wild animals, increasing the potential for cross-species infection.

During PRV infection in pigs, the virus initially replicates in the respiratory epithelial cells (28–31). Subsequently breaching the basement membrane with the assistance of infected white blood cells, infiltrating the deep connective tissue, and entering the bloodstream and local lymph nodes, leading to viremia associated with peripheral blood monocytes (32, 33). PRV can also undergo secondary replication in the uterus of pregnant sows, where infected monocytes cross the maternal vascular endothelial barrier to reach the uterus (34, 35). In pregnant sows, early-stage PRV infection results in widespread endometrial infection causing fetal membrane separation and leading to abortion or fetal resorption. During the middle and late stages of pregnancy, the virus can cross the placenta and infect the fetus, resulting in spontaneous fetal abortion or stillbirth (31, 36). PRV exhibits broad tissue tropism and neurotropism, allowing it to establish latency in the central nervous system (37, 38). During initial infection, the virus invades the peripheral nervous system through nerve endings, including the trigeminal ganglion and afferent nerve fibers of the olfactory bulb. It then reaches the ganglia, where it remains latent via retrograde axonal transport (38, 39). Clinically recovered pigs typically exhibit no apparent symptoms, but the latent virus can reactivate under stressful conditions (40).

Research indicates that pigs exhibit substantial age-related differences in susceptibility to PRV. Juvenile pigs mainly develop fatal infections with severe neurological symptoms, whereas adult pigs (>1 year old) typically experience respiratory symptoms or subclinical infections (41). Notably, cases of severe central nervous system lesions, respiratory disease, and mortality due to PRV infection are relatively uncommon in pigs older than 6 weeks (41). Several studies suggest that PRV variant strains have likely overcome the age-related resistance barrier in pig populations. Yang et al. demonstrated that the PRV variant HN1201 exhibits greater pathogenicity in pigs aged 35–127 days compared to the classical strain Fa (42). Zhou et al. isolated two lethal PRV variants (PRV-GD and PRV-JM), both of which induced severe respiratory distress and neurological symptoms in 60-day-old pigs, resulting in 100% mortality (3/3) (43). More recently, Chen et al. reported that the HeN21 strain caused severe respiratory and neurological symptoms in 90-day-old pigs, with all three infected pigs succumbing to the disease (44). This study further suggested that the emergence of HeN21 may indicate the presence of a more virulent PRV strain in China (44).

In this study, we report the bioinformatics and pathogenicity analysis of a PRV strain, GDWS2, isolated from an infected farm in Guangdong Province, China, which had been vaccinated with the Bartha-K61 vaccine. Our findings reveal multiple genomic variations in the GDWS2 strain and its genome contains recombination events with PRV variants, Bartha-k61, or genotype I strains. Compared with the variant strain JM, which was isolated in China in 2021, GDWS2 causes more severe respiratory and neurological signs and increased viral shedding in 90-day-old pigs. The presence of the GDWS2 strain may suggest the re-emergence of a more virulent PRV strain in China.

## 2 Materials and methods

### 2.1 PRV strains and cell culture

In 2024, a PRV outbreak occurred on a fattening pig farm in Guangdong Province, China, which had been vaccinated with the Bartha-K61 vaccine. A pig (40 kg, 90-day-old, male, Duroc × Landrace × Large White crossbred) succumbed to the disease after exhibiting high fever and neurological symptoms. Post-mortem examination revealed severe meningeal congestion and pulmonary hemorrhage (Supplementary Figure S1). The nucleic acid from the brain tissue of this pig was collected. Only PRV was detected in the tissue, and the PRV GDWS2 strain was subsequently isolated. The previously collected PRV strains include one variant strain (JM strain, OK338077), one classic strain (Fa strain, KM189913), and one vaccine strain (Bartha-k61 strain, JF797217), which were used as control strains (45). The PRV-JM strain belongs to the genotype II variant strain. After infecting 60-day-old pigs, it can cause severe neurological and respiratory symptoms, and the mortality rate is as high as 100%. Characteristics of this strain have been described in the literature (43). Additionally, 23 genome sequences were downloaded from NCBI for bioinformatics analysis. Information on the reference strains is provided in Table 1. The cells used in this study (preserved in our laboratory)—PK-15 (ATCC CCL-33), MRC-145 (ATCC CRL-12231), VERO (ATCC CCL-81) and BHK-21 (ATCC CCL-10)—were all cultured in Dulbecco’s modified Eagle medium (DMEM; Gibco, USA) supplemented with 10% fetal bovine serum (Gibco, USA) and maintained in medium containing 2% fetal bovine serum. PAM cells (derived from the lungs of 5-week-old specific pathogen-free pigs) were preserved in the laboratory and maintained in 1640S medium (Gibco, USA) with 2% fetal bovine serum.

**Table 1:** Information of PRV reference strains used in this study.

Virus name	Discovery time	Species	Origin	Accession No.	Genotype
HeN21	2023	Swine	China	OP906304	II
FJ	2022	Swine	China	MW286330	II
JM	2021	Swine	China	OK338077	II
GD	2021	Swine	China	OK338076	II
JS-2020	2020	Swine	China	OR271601	II
SX1911	2019	Swine	China	OP376823	II
HuBXY	2018	Swine	China	MT468549	II
LA	2017	Swine	China	KU552118	II
GXGG	1016	Swine	China	OP605538	II
GD0304	2016	Swine	China	MH582511	II
HEN1	2015	Swine	China	KP098534	II
GDYH	2014	Swine	China	MT197597	II
TJ	2012	Swine	China	KJ789182	II
HNX	2012	Swine	China	KM189912	II
JS-2012	2012	Swine	China	KP257591	II
XJ	2012	Swine	China	MW893682	II
Fa	2001	Swine	China	KM189913	II
EA	1999	Swine	China	KU315430	II
SC	1987	Swine	China	KT809429	II
FB	1986	Swine	China	ON005002	II
Kaplan	2014	Swine	Hungary	KJ717942	I
Becker	2011	Swine	USA	JF797219	I
Bartha	2011	Swine	Hungary	JF797217	I

Virus isolation was carried out following the method described in reference (46). After tissue samples were cut into small pieces, phosphate-buffered saline (PBS) was added for homogenization. The sample was frozen and thawed three times at −80°C. After centrifugation at 12,000 rpm for 5 min, the supernatant was filtered through a 0.22-μm membrane and inoculated into PK-15 cells. Following 1-h incubation at 37°C, the virus solution was discarded, and maintenance medium was added (45). Observe it under the microscope every day. Upon the occurrence of obvious cytopathic effects such as swelling, shedding, aggregation, and syncytia formation, cell supernatant was collected and subjected to three freeze–thaw cycles. After three blind passages, plaque purification was performed. The virus solution was serially diluted in a tenfold gradient with DMEM. Then, 300 μL of the diluted solution was taken to infect the PK-15 cells in 6-well plates. After 1 h of infection, maintenance medium comprising 1% low-melting-point agarose at 37°C was added, and the plates were incubated upside down for 3–5 days. Plaques were stained with neutral red (Beyotime, China), marked under a microscope, and collected using a 1000-μL pipette tip. The collected plaques were suspended in DMEM and subjected to three freeze–thaw cycles. After dilution with DMEM, the purification step was repeated three times to obtain the purified virus solution. Subsequently, the purified virus solution was diluted at a ratio of 1:9 and propagated in PK-15 cells, yielding 30 mL of the final virus solution. Finally, the virus solution was serially diluted in a tenfold gradient with DMEM, and then 300 μL of the diluted solution was inoculated into PK-15 cells for indirect immunofluorescence detection of the gE protein. The presence of a fluorescence signal indicated successful isolation (47). The morphology of the isolated strain was observed using a scanning electron microscope.

### 2.2 Biological characteristics of the virus

To assess the virulence of the virus in cells, we first determined the 50% tissue culture infective dose (TCID50) for each PRV strain in PK-15 cells (48). Simultaneously, the one-step growth curve method was employed to infect PK-15 cells at a multiplicity of infection (MOI) of 0.01. Samples were collected at different time points to determine the viral titer, and a viral growth curve was plotted. Herpesviruses exhibit a broad host range and can infect various mammalian species (49, 50). To verify the broad tropism of the isolated strain (46), the viruses were inoculated into 6-well plates (Thermo Fisher, USA) seeded with 90% confluent cells of various types (PK-15, MRC145, VERO, and BHK) at a MOI of 0.1 and incubated at 37°C. The cytopathic effect was observed 36 h post-infection (44). To evaluate the virulence differences among the different virus strains, plaque assay was performed (51). Specifically, each virus strain was inoculated into PK-15 cells in 6-well plates (*n* = 6) at an MOI of 0.01. After 1 h of infection, maintenance medium comprising 1% low-melting-point agarose at 37°C was added, and the plates were incubated upside down for 3 days. Staining was performed (4% paraformaldehyde and 0.1% crystal violet) (51), and the plaques were visualized after washing the cells with tap water. Subsequently, 10 intact plaques were randomly selected from each replicate sample and their areas were measured using ImageJ software. The average plaque area of each replicate sample was determined.

### 2.3 Whole-genome sequencing and bioinformatics analysis

Whole-genome sequencing was performed using a previously described method (52). Viral DNA was extracted using a commercial DNA/RNA extraction kit (Vazyme, Nanjing, China), and the quality and quantity of the extracted DNA were assessed via agarose gel electrophoresis and a Qubit^®^ 2.0 fluorometer (Thermo Scientific, USA). A sequencing library was prepared using Nextera™ DNA Flex Library Preparation Kit (Illumina, CA) according to the manufacturer’s instructions, and sequencing was performed using the MGI platform. The sequencing read length was 150 bp at each end. After filtering low-quality reads, clean data were obtained. SPAdes (version 3.15.4) was used for genome assembly, resulting in a 1,42,247-bp genome sequence of GDWS2 with a G + C content of 73.68%. The genome was annotated using the RAST server (53). Nucleotide and amino acid similarities were calculated using MegAlign, and data visualization was performed using GraphPad Prism 8. Gene sequence alignments were displayed using GeneDoc (version 2.7). Phylogenetic analysis was conducted using MEGA software (version 27) (54), based on the nucleotide sequence alignments of gC, gB, gD, gE, TK, and the whole genome. The bootstrap value was set to 1,000, and the phylogenetic tree was visualized using iTOL tool (55). Recombination events were considered reliable if they were detected by at least three of the seven selected algorithms (RDP, GENECONV, BootScan, Maxchi, Chimaera, SiScan, and 3Seq) with a significance level of *p* < 0.01 (56).

### 2.4 Animal experiments

Animal experiments were conducted at the Institute of Guangdong Wens Food Group (Yunfu, China) and were approved by the school’s ethics committee. The approval numbers for the rabbit and pig experiments are (approval IDs: fosu#088625 and fosu#088644, respectively). The experiments were conducted in strict accordance with the provisions of the “Regulations on the Administration of Laboratory Animals in China” and the “Regulations on the Administration of Laboratory Animals in Guangdong Province.”

To assess the virulence of the virus, 66 New Zealand white rabbits (70-day-old, specific pathogen-free level, male), each weighing 2 kg, were randomly categorized into 11 groups (Groups P1–P11), with 6 rabbits per group. Rabbits in Groups P1–P5 were subcutaneously injected at the back of the neck with the GDWS2 strain virus solution at doses of 1 × 10^5^, 1 × 10^4^, 1 × 10^3^, 1 × 10^2^, and 1 × 10^1^ TCID50, respectively. Furthermore, rabbits in Groups P6–P10 were injected with the JM strain virus solution at the same doses (rabbit models and routes of infection refer to the Chinese National Standard “Diagnostic method for pseudorabies GB/T 18461-2018”). Following the challenge, rabbit deaths were recorded, a survival curve was plotted, and the 50% lethal dose (LD50) was calculated. Heart, liver, spleen, lung, and brain tissues were collected, and 0.2 g of tissue was homogenized in 800 μL of PBS and centrifuged. Then, 400 μL of the supernatant was used for viral DNA extraction. Nucleic acid was extracted using a commercial kit (Bioer, BSC16, China) and an automatic nucleic acid extractor (Bioer, NPA-16H, China). Quantitative polymerase chain reaction (qPCR) was used to detect viral DNA and calculate the viral load using a specific primer (PRV-gE-F:5′-TGCCGCGGCTCCGGCGCGAG-3′, PRV-gE-R: 5′-CGCACCTTCGCCCCGAGCAC-3′) with reference to previous literature (57). The gE plasmid was used to evaluate DNA copy number using the formula *y* = 3.6741x + 47.883. Hematoxylin–eosin staining was performed for histological examination of the collected tissues.

To evaluate the virulence of PRV strains in fattening pigs, the experiment used 90-day-old pigs (40 kg, 90-day-old, male, Duroc × Landrace × Large White crossbred) by categorizing into three groups (DMEM, GDWS2, and JM groups) with 6 pigs per group. Before the challenge, pig blood as well as anal, oral, pharyngeal, and nasal swabs were collected to test for PRV, PRRSV, PCV, CSFV, and ASFV viral DNA and antibodies against PRV gE and gB, ensuring all samples were negative. The GDWS2 strain (2 × 10^7^ TCID50), JM strain (2 × 10^7^ TCID50) and DMEM (2 mL) were injected into the neck muscles of pigs [the route of infection refers to (58)]. The experiment lasted for 15 days. After infection, rectal temperature was measured every other day, and blood, nasal, oral, and anal swabs were collected for qPCR detection of viral DNA copy numbers. At the conclusion of the experiment, heart, liver, spleen, lung, kidney, brain, cerebellum, pons, medulla oblongata, spinal cord, sciatic nerve, hilar lymph nodes, splenic hilar lymph nodes, mandibular lymph nodes, and tonsils were collected for viral DNA detection, and DNA copy numbers were calculated. Hematoxylin–eosin staining was performed for histological examination of the collected tissues. Histopathological scoring of brain tissues was conducted as described in (49) to reflect the extent of pathological damage.

### 2.5 Statistical analysis

Statistical analysis was performed using the independent sample t-test via IBM SPSS Statistics 27. Data were presented as mean ± standard error of the mean. Images were generated using GraphPad Prism 9.5 and processed using Adobe Photoshop 2024.

## 3 Results

### 3.1 Virus isolation and biological characteristics of the isolated strain

**Figure 1:**
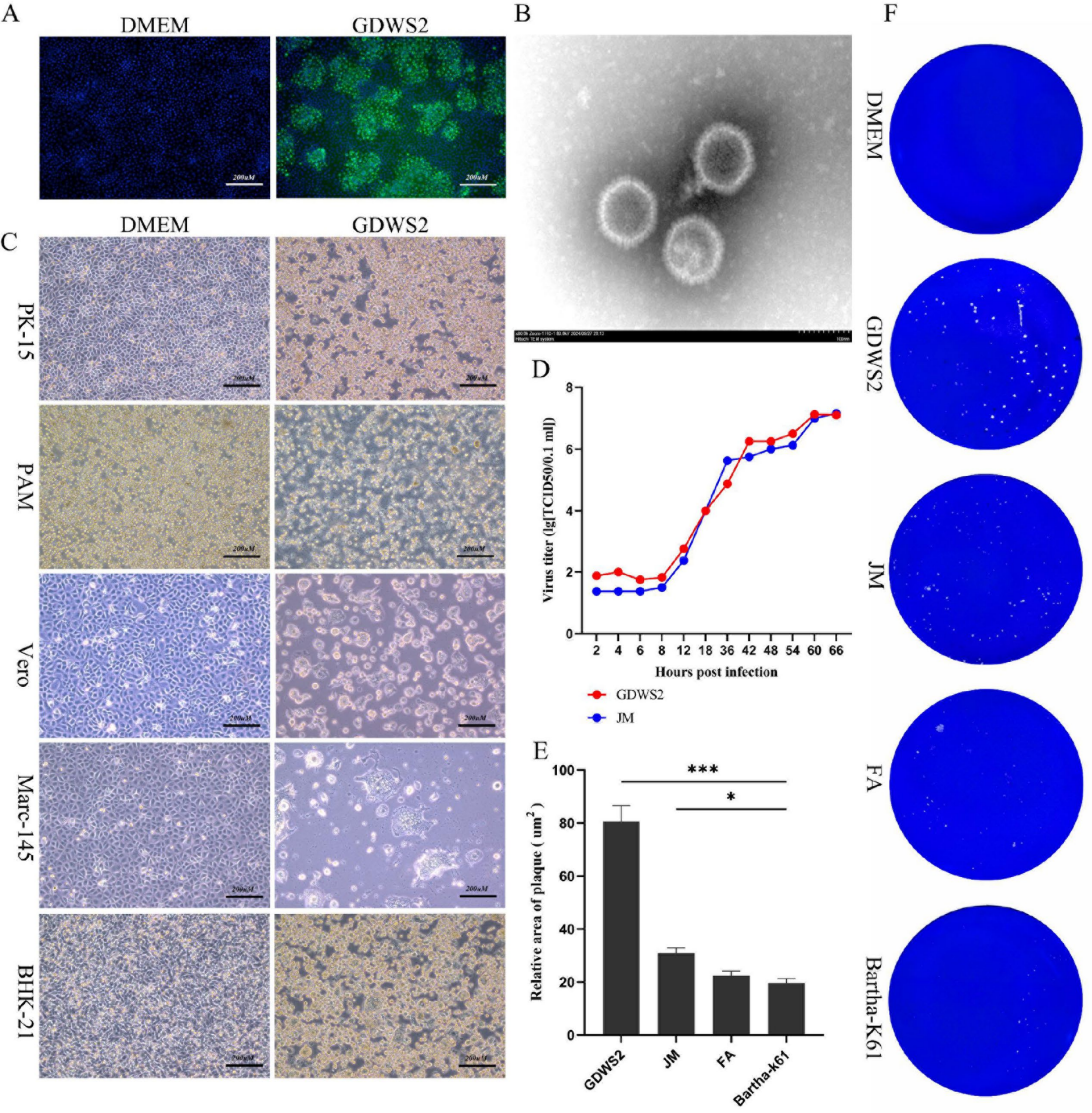
Isolation, identification of the virus and biological characteristics of the virus strain. **(A)** Indirect immunofluorescence analysis of PK-15 cells infected by GDWS2 strain. The green signal is gE antibody. **(B)** Morphological characteristics of GDWS2 strain under an electron microscope (bar = 100 nm). **(C)** Cytopathic effect of GDWS2 strain on cells from different sources. **(D)** One-step growth curves of different strains. **(E)** Sizes of plaques caused by different PRV strains on PK-15 cells quantified using the ImageJ software (*n* = 6). **(F)** Plaque morphologies of different PRV strains on PK-15 cells. The significance level was set at *p* < 0.05 (∗), *p* < 0.01 (∗∗), and *p* < 0.001 (∗∗∗).

Brain tissue from the deceased pig was collected, homogenized, and centrifuged. Bacteria were removed via filtration with a 0.22-μm membrane, and the sample was inoculated into PK-15 cells. Once lesions appeared, the supernatant was collected, and the cells were re-infected. Following plaque purification, the presence of the PRV gE protein signal in infected cells was confirmed through indirect immunofluorescence (Figure 1A). The morphology of the isolated strain was observed using an electron microscope (Figure 1B). This led to the isolation of a PRV strain from the brain tissue, named GDWS2. To study the biological characteristics of the PRV GDWS2 strain, we analyzed its cell tropism. Pathogenicity tests of various cells (Figure 1C) showed that GDWS2 infection induced cell lesions such as swelling, shedding, aggregation, and syncytia formation in porcine-derived cells (PK-15 and PAM), monkey-derived cells (VERO and Marc-145) and mouse-derived cells (BHK-21). As shown in the one-step growth curve (Figure 1D), during the early infection phase (2–8 h), the viral titer of the GDWS2 strain was higher than that of the JM strain (a recently discovered PRV variant). GDWS2 exhibited the fastest growth between 12 and 42 h, reaching a peak titer of 1 × 10^7^ TCID50/0.1 mL at 60–66 h. In addition, plaque analysis of GDWS2, JM, Fa, and Bartha-K61 strains revealed that the plaque size of GDWS2 strain was significantly larger than those of JM, Fa, and Bartha-K61 strains (*p* < 0.05) (Figures 1E,F).

### 3.2 Bioinformatics analysis

**Figure 2:**
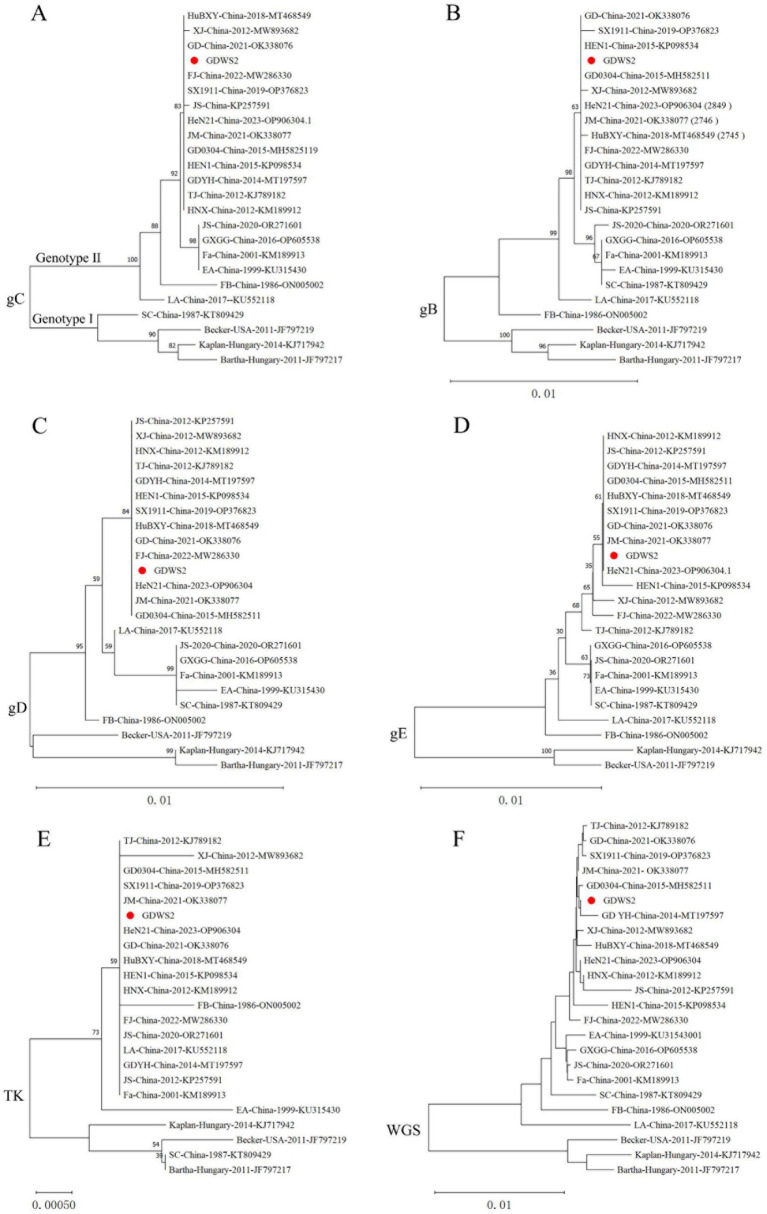
Phylogenetic analysis of different PRV strains. **(A)** A phylogenetic tree generated based on the nucleotide sequences of the *gC* gene. **(B)** A phylogenetic tree generated based on the nucleotide sequences of the *gB* gene. **(C)** A phylogenetic tree generated based on the nucleotide sequences of the *gD* gene. **(D)** A phylogenetic tree generated based on the nucleotide sequences of the *gE* gene. **(E)** A phylogenetic tree generated based on the nucleotide sequences of the *TK* gene. **(F)** A phylogenetic tree generated based on the whole-genome sequences (*WGS*).

According to the phylogenetic analysis of the *gC* gene and the genetic evolution analysis of the major virulence genes and the whole genome (Figure 2), the GDWS2 strain was identified as genotype II and belonged to the same clade as the PRV variant strains discovered in China after 2011 (Figure 2A). Phylogenetic analysis of the *gB* gene revealed that it was closely related to FJ and GD0304 strains (Figure 2B). Moreover, the phylogenetic analysis of the *gD*, *gE*, and thymidine kinase (*TK*) genes showed that they all had a close relationship with the variant strains isolated in recent years, including the HeN21, GD, and JM strains. Additionally, *gD* was closely related to the FJ strain (Figures 2C–E). The phylogenetic analysis of the whole-genome sequencing also revealed that the GDWS2 strain, along with the GDYH and GD0304 strains discovered in Guangdong Province, appeared in a separate branch and had the closest relationship (Figure 2F).

**Figure 3:**
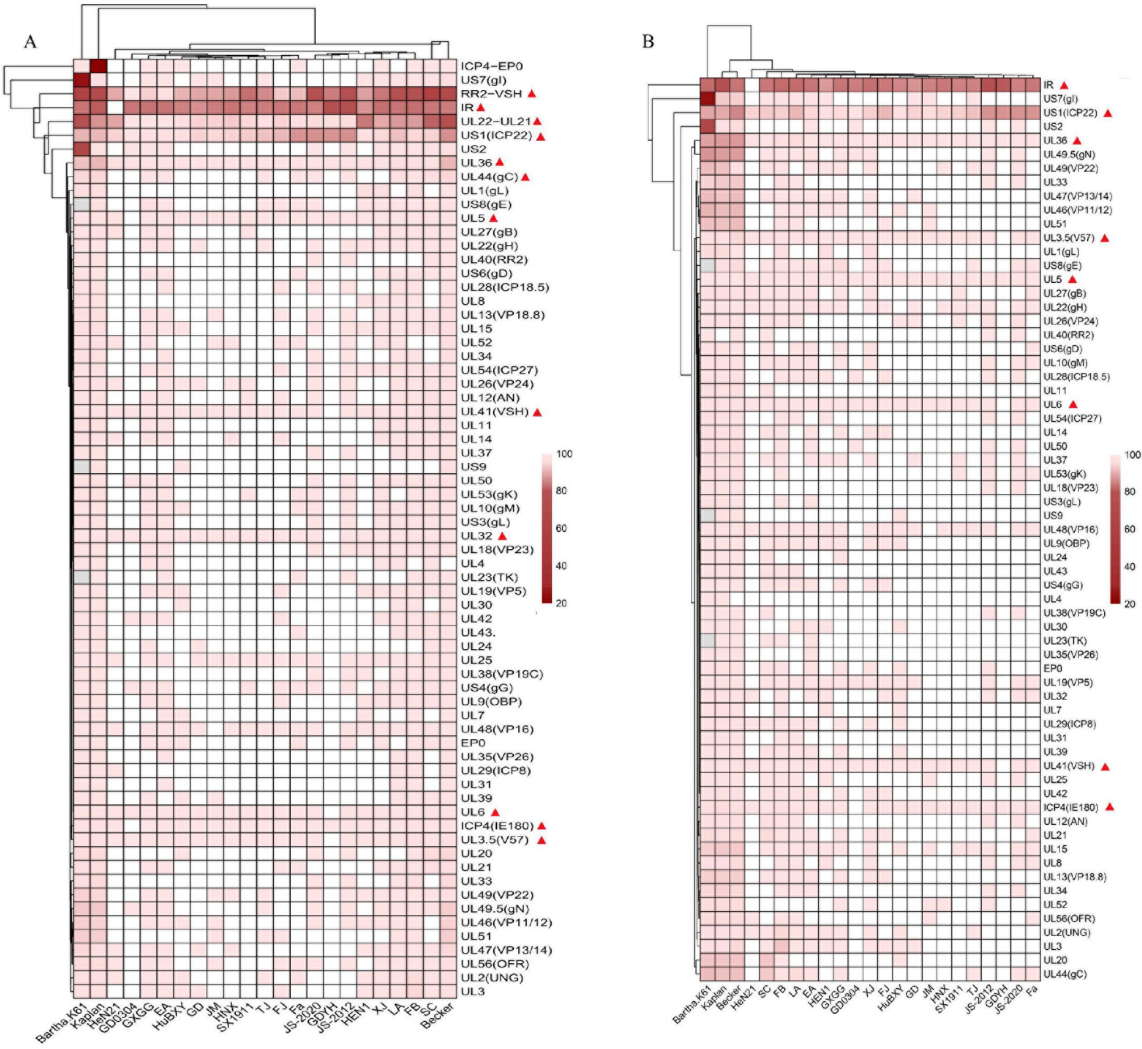
Genome divergence between PRV strain GDWS2 and the reference strains. **(A)** Nucleotides; **(B)** Amino acids. The divergence is depicted in colors ranging from dark (the highest) to bright (the lowest), and gene deletions are represented in gray.

**Figure 4:**
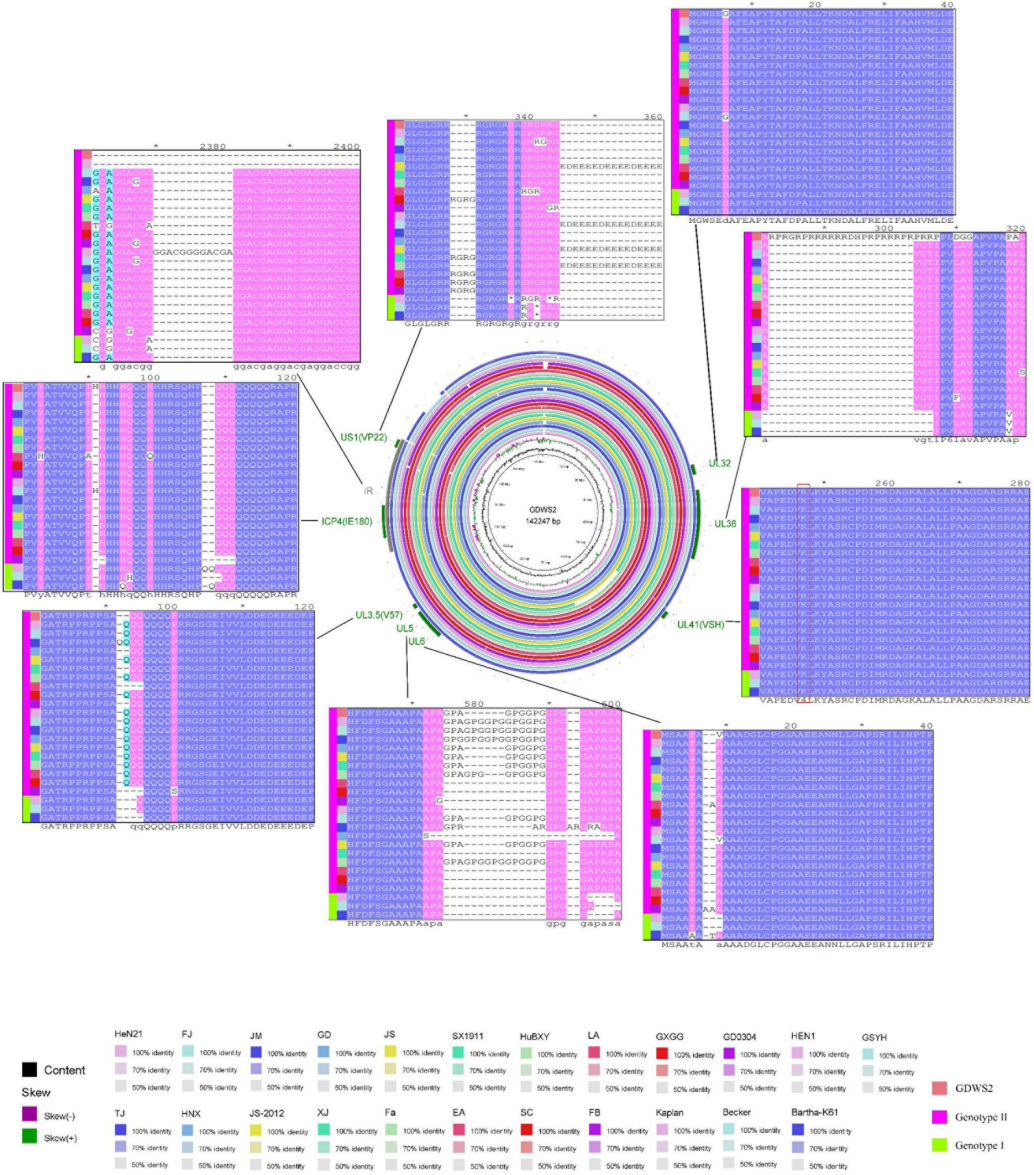
Circle map showing complete genome sequence alignments of GDWS2 and strains.

We further analyzed the GDWS2 sequence by conducting similarity analysis of 65 proteins and 3 noncoding regions between GDWS2 with 23 reference strains. A heatmap was constructed based on the sequence similarities between GDWS2 and the reference strains (Figure 3). The results showed varying degrees of nucleotide and amino acid differences between GDWS2 and the reference strains, particularly when compared with genotype I strains. In terms of nucleotide homology within the protein-coding regions (Figure 3A), *UL36* and *US1* (ICP22) differed between GDWS2 and all reference strains, whereas *UL3.5*, *UL32*, *UL41* (VSH), and *UL6* were identical between GDWS2 and the GD YH strain. Additionally, *ICP4* (IE180) and *UL5* were identical between GDWS2 and GD0304 strains, but they differed between GDWS2 and other reference strains. Similar patterns were observed in amino acid homology analysis; however, UL5 differed between GDWS2 and GD0304 strains at the protein level (Figure 3B). Regarding the nucleotide sequences of *RR2* to *VSH* and *UL22* to *UL21* in the noncoding region, the GDWS2 strain showed the lowest similarity to the reference strains, whereas the IR region of GDWS2 was identical to that of HeN21 but differed from those of other strains (Figure 3A). Further alignment of amino acid sequences in regions with considerable differences revealed insertions or variations in US1, UL36, and UL5 between GDWS2 and the reference strains. UL3.5, UL6, and UL41 shared identical amino acid sequences between GDWS2 and GD YH strains, whereas ICP4 was identical between GDWS2 and GD0304 strains. IR and HeN21 exhibited a section of amino acid deletion (Figure 4). These findings were consistent with those of amino acid similarity.

**Figure 5:**
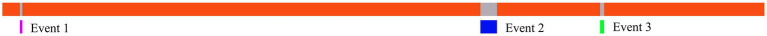
Analysis of the recombination of different PRV strains. Analysis of the recombination of the entire genome of PRV GDWS2 (in red). The recombination events predicted by RDP4 are presented in purple (SX1911 + Kaplant), blue (Becker + Bartha), and green (JS-2020 + Bartha).

Recombination analysis using RDP4 revealed multiple recombination events in the GDWS2 strain. These include event 1: recombination between a PRV variant strain and a classic strain (SX1911 + Kaplant), event 2: recombination between a PRV classic strain and a vaccine strain (Becker + Bartha), and event 3: recombination between a PRV variant strain and a vaccine strain (JS-2020 + Bartha) (Figure 5). These recombination events primarily occurred in protein-coding regions, such as event 1 (UL54), event 2 (noncoding region + IR), and event 3 (partial US8, US9, and noncoding regions).

### 3.3 Study on the virulence of PRV GDWS2 strain to rabbits and fattening pigs

**Figure 6:**
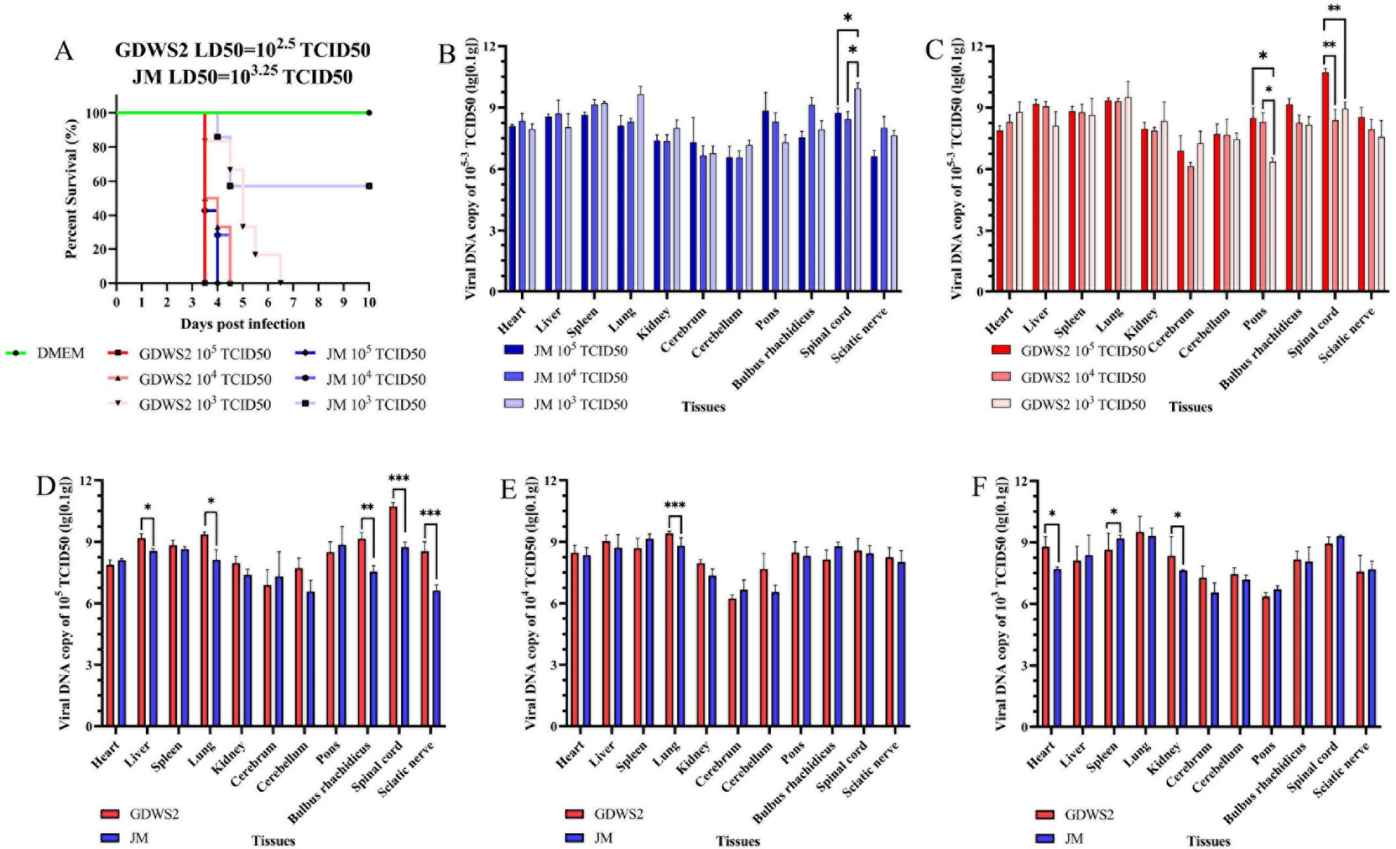
Evaluation of viral virulence by LD50 in rabbit models. **(A)** The survival curve of experimental ribbit inoculated with different PRV strains or DMEM. **(B)** Detection of viral DNA in rabbit tissues infected with different doses of the JM strain by fluorescence quantification. **(C)** Detection of viral DNA in rabbit tissues infected with different doses of the GDWS2 strain by fluorescence quantification. **(D)** Comparison of viral DNA in various organs of different strains at an infection dose of 1 × 10^5^ TCID50. **(E)** Comparison of viral DNA in various organs of different strains at an infection dose of 1 × 10^4^ TCID50. **(F)** Comparison of viral DNA in various organs of different strains at an infection dose of 1 × 10^3^ TCID50. The significance level was set at *p* < 0.05 (∗), *p* < 0.01 (∗∗), and *p* < 0.001 (∗∗∗).

To study the virulence of the isolated PRV GDWS2 strain, we subcutaneously inoculated New Zealand white rabbits with either the GDWS2 strain, JM strain or DMEM at different infection doses to determine the LD50 of the PRV strains. Following infection with the two strains at doses of 1 × 10^5^, 1 × 10^4^, 1 × 10^3^ TCID50, neurological symptoms (such as skin scratching) and death occurred within 3.5–6.5 d (Figure 6A). All rabbits in the GDWS2 experimental group died at a dose of 1 × 10^5^, 1 × 10^4^, 1 × 10^3^ TCID50. In the JM experimental group, all rabbits died at a dose of 1 × 10^5^, 1 × 10^4^ TCID50, while two rabbits died at a dose of 1 × 10^3^ TCID50. The calculated LD50 for the GDWS2 strain in rabbits was 1 × 10^2.5^ TCID50, and for the JM strain, it was 1 × 10^3.25^ TCID50 (Figure 6A).

Viral DNA was detected in various organ tissues of deceased rabbits (Figures 6B,C), with the highest viral copy numbers found in the lungs, pons, medulla oblongata, and spinal cord. The viral DNA load differed across tissues depending on the infection dose. In the GDWS2 strain, viral load in the pons was significantly higher at infection doses of 1 × 10^5^ and 1 × 10^4^ TCID50 compared with 1 × 10^3^ TCID50, and the spinal cord viral load at 1 × 10^5^ TCID50 was significantly higher than at 1 × 10^4^ and 1 × 10^3^ TCID50 (Figure 6B). For the JM strain, spinal cord viral load at the 1 × 10^3^ TCID50 infection dose was significantly higher than at 1 × 10^4^ and 1 × 10^3^ TCID50, with no significant differences observed in other tissues (Figure 6C). Comparing the two strains at the infection dose of 1 × 10^5^ TCID50, the GDWS2 strain exhibited significantly higher viral loads in the liver, lungs, medulla oblongata, spinal cord, and sciatic nerve compared with the JM strain (Figure 6D). At an infection dose of 1 × 10^4^ TCID50, the viral load in the lungs of the GDWS2 strain was higher than that of the JM strain (Figure 6E). At 1 × 10^3^ TCID50, the viral load in the heart, spleen, and kidneys of the GDWS2 strain was significantly higher than in the JM strain (Figure 6F), with no significant differences in other tissues. Histological examination of rabbits infected at a 1 × 10^5^ TCID50 dose showed varying degrees of tissue damage (Supplementary Figure S2). The heart showed inflammatory cell infiltration and eosinophilic inclusions, while the spleen exhibited hemorrhage. In the kidneys, glomerular swelling and renal tubular atrophy were observed. In the brain, cerebellum, pons and medulla oblongata, nerve cell lysis, vascular sheath formation, vascular dilation and congestion, glial cell lesions, nerve cell phagocytosis, tissue loosening, indistinct Purkinje cells, and Purkinje cell disappearance were noted.

**Figure 7:**
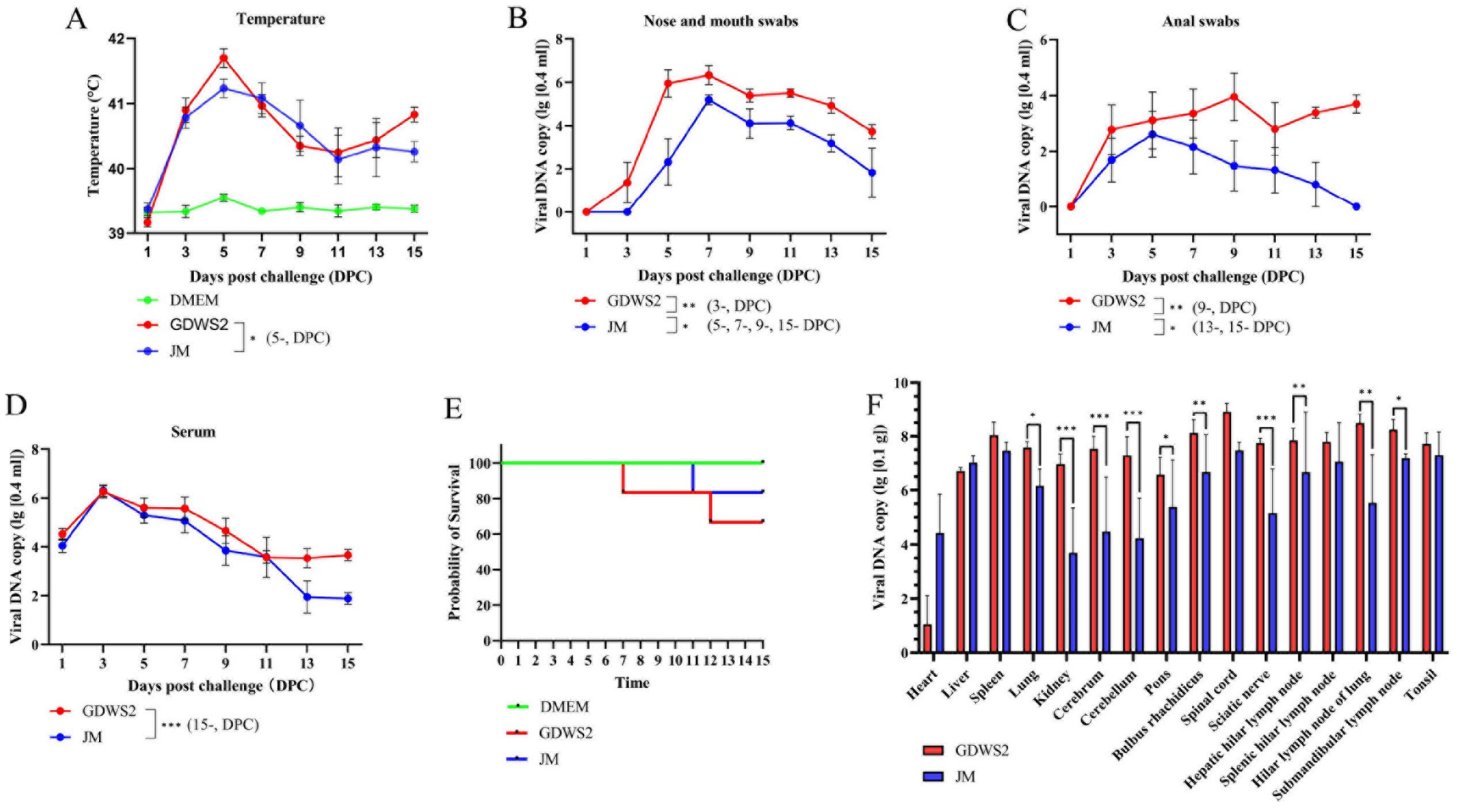
Evaluation of viral virulence using pig models. **(A)** Survival curve of experimental pigs inoculated with different PRV strains or DMEM. **(B)** Rectal temperature of pigs inoculated with different PRV strains or DMEM at different days post inoculation. **(C)** Detection of viral DNA in the nose and mouth swabs collected from pigs inoculated with different PRV strains at different days post inoculation by real-time quantitative PCR. **(D)** Detection of viral DNA in anal swabs collected from pigs inoculated with different PRV strains at different days after inoculation by real-time quantitative PCR. **(E)** Detection of viral DNA in blood collected from pigs inoculated with different PRV strains at different days after inoculation by real-time quantitative PCR. **(F)** Detection of viral DNA in different pig tissues by real-time quantitative PCR. The significance level was set at *p* < 0.05 (∗), *p* < 0.01 (∗∗), and *p* < 0.001 (∗∗∗).

We tested the virulence of GDWS2 and JM strains in fattening pigs (Figure 7). On day 5 of the experiment, pigs in the GDWS2 group showed more severe respiratory symptoms, such as facial swelling, abdominal breathing, and purulent nasal discharge as well as neurological symptoms, including inability to stand, unsteady gait, and difficulty eating. Two pigs in the GDWS2 group died on days 7 and 12, respectively, whereas one pig in the JM group died on day 11 (Figure 7E). Additionally, all pigs showed an increase in rectal temperature. On day 5, body temperatures in the GDWS2 group were significantly higher than those in the JM group (Figure 7A). Viral shedding was monitored, revealing that the viral copy numbers in nasal, oral, and anal swabs were consistently higher in the GDWS2 group than in the JM group (Figures 7B,C). Specifically, viral copy numbers in nasal and oral swabs were significantly higher in the GDWS2 group than in the JM group on days 3, 5, 7, 9, and 15 post-infection. Furthermore, viral copy numbers in anal swabs were significantly higher in the GDWS2 group on days 9, 13, and 15 post-infection. Although viral copy numbers in the serum were similar between the two groups, on day 15 post-infection, the GDWS2 strain showed significantly higher viral copy numbers than the JM strain (Figure 7D). Post-challenge, viral copy numbers in the spleen, lungs, kidneys, brain, cerebellum, pons, medulla oblongata, sciatic nerve, submandibular lymph nodes, liver, and hilar lymph nodes of the GDWS2 group were significantly higher than those of the JM group, with the spinal cord displaying the highest viral copy number (Figure 7F). Histological examination revealed tissue lesions similar to those observed in the rabbit model (Supplementary Figure S3). Lymph nodes and tonsils showed enlarged follicles, disordered structures, and vascular congestion or hemorrhage (Supplementary Figures S4A–F). The brain histopathological score (Supplementary Table S1) indicated that the numbers of lesions in the brain, cerebellum, pons, and medulla oblongata were significantly higher in the GDWS2 group than in the JM group (*p* < 0.05).

## 4 Discussion

Since the 1980s, PRV has spread globally owing to international trade of animal products. Countries such as Germany, Switzerland, the United Kingdom, and Canada have successfully eradicated PR through strict disease-control measures, eradication programs, and extensive use of the Bartha-k61 vaccine (1). However, PR remains endemic in regions practicing intensive pig farming, such as Eastern Europe, Southeastern Europe, Latin America, Africa, and parts of Asia (1, 59). Although multiple studies have reported the development of candidate vaccines against PRV, demonstrating considerable protective effects in mouse and pig models (16, 19–25), the global epidemiological situation of PRV remains complex and concerning. Wild boars as reservoir hosts of PRV pose a significant threat for the reintroduction of the virus into disease-free areas or cross-species transmission. Between 2011 and 2015, Germany tested 108,748 and northwestern Italy tested 1,425 wild boar serum samples, respectively, with PRV antibody positivity rates of 12.09 and 30.39%, respectively (60, 61). Additionally, the Campania region (Italy) reported cases of hunting dogs contracting Pseudorabies after contact with wild boars (27). More alarmingly, emerging PRV variants not only threaten pig populations but can also infect humans, causing blindness, encephalitis, and even death (62). These findings indicate the high pathogenicity and cross-species transmission potential of these variants, further emphasizing the significant public health threat posed by PRV.

Since 2011, multiple pig farms in China that vaccinated against PRV with the Bartha-K61 strain have experienced outbreaks caused by PRV variant strains (8, 13). These variants have quickly became the dominant epidemic strains, leading to substantial economic losses in the swine industry. Studies show that compared to the classical Fa strain, PRV variants such as HN1201 exhibit increased pathogenicity in pigs (42). However, PRV susceptibility varies significantly with age—variant strains generally do not induce severe respiratory or neurological symptoms, nor do they cause mortality in older pigs (≥90 days old) (18). This was demonstrated in studies where the SMX-2012 variant, which causes fatal respiratory disease in piglets, only triggered mild respiratory symptoms in 90-day-old pigs (18). Notably, our study found that 90-day-old pigs inoculated with GDWS2 developed severe respiratory and neurological symptoms, with cases of mortality. Importantly, the farm where GDWS2 was isolated had implemented Bartha-K61 vaccination, yet the vaccine failed to confer complete protection—resembling the initial variant outbreaks in 2011 (13, 14). To assess GDWS2 virulence, *in vitro* experiments showed that this strain formed significantly larger plaques in PK-15 cells than other strains. Additionally, its viral titer in the early growth phase was higher than that of other variant strains isolated in China. These findings collectively suggest that the virulence of the GDWS2 strain has undergone significant alterations.

High-throughput sequencing technology has greatly advanced PRV whole-genome studies, leading to the complete sequencing of multiple PRV variant strains. This study analyzed 23 representative strains, including classical strains predating 2011, variant strains from 2012 to 2023, and strains prevalent in other countries. Phylogenetic analysis of the *gC* gene reveals that the GDWS2 strain belongs to genotype II, forming an independent evolutionary branch alongside PRV variants isolated in China after 2011. Notably, this strain shares high homology in the *gD*, *gE*, and *TK* genes with strains such as HeN21, GD, and JM—primary pathogens responsible for severe respiratory symptoms, neurological signs, and mortality in pigs aged 60–90 days (43, 44). Additionally, GDWS2 exhibits significant nucleotide sequence variations in US1 (ICP22) UL36, and as well as amino acid differences in UL5, compared to reference strains. The pUL36 protein, encoded by UL36 is known to facilitate viral particle assembly and neuroinvasion (6), while ICP22, encoded by US1 regulates early host cell infection and immune evasion (7). The UL5 gene product also plays a key role in initiating viral DNA replication (5). These functional gene variations may underlie the enhanced virulence of GDWS2. Furthermore, GDWS2 shares specific amino acid arrangements in UL3.5, UL6, UL41, and ICP4 with GDYH and GD0304 strains isolated in Guangdong—suggesting that PRV variants emerging in specific regions likely originate from a common ancestral strain.

Notably, the IR nucleotide sequence of the GDWS2 strain is completely identical to that of the HeN21 strain. Previous studies have shown that the IR region of HeN21 participates in multiple recombination events (44), suggesting that GDWS2 may also be a recombinant. To test this hypothesis, we conducted a recombination analysis, which revealed that GDWS2 is a natural recombinant between a PRV heterologous strain and either the Bartha strain or a genotype I strain. Recombination plays a crucial role in viral genome evolution, and previous studies have documented recombination events among different PRV strains. For instance, the GXLB2 strain resulted from recombination between a PRV variant strain and the Bartha-K61 vaccine strain (63), and the JSY13 strain emerged from recombination between the genotype I Bartha-K61 vaccine strain and a genotype II PRV variant strain (64). Given the epidemiological background of the GDWS2 strain, we speculate that these recombination events may be linked to immune pressure from the prolonged use of the attenuated Bartha-K61 vaccine. The extensive application of live vaccines in China likely increases the probability of recombination between vaccine and wild strains. Notably, previous studies indicate that recombination in the gB gene can significantly alter immunogenicity, as observed in the JS-2012 strain compared to the Bartha-K61 vaccine strain (65). However, the precise impact of recombination on the biological characteristics of the GDWS2 strain requires further in-depth investigation.

As a member of the Herpesviridae family, PRV has a broad host range, infecting various mammals including pigs (49), rabbits (50), rhesus monkeys (66), and hamsters (67). Mice and rabbits are commonly used as PRV animal models in experimental studies (8). In this study, *in vitro* experiments confirmed that the GDWS2 strain could infect porcine, monkey, and murine-derived cells, inducing significant cytopathic effects, thereby verifying its broad host tropism. Using the highly pathogenic genotype II variant strain JM as a reference, we then evaluated the virulence of GDWS2 in two animal models. In the LD50 test using rabbits, the LD50 of the GDWS2 strain was 1 × 10^2.5^ TCID50—significantly lower than that of the JM strain (1 × 10^3.25^ TCID50) indicating greater virulence. Viral DNA copy numbers varied significantly across organ tissues in deceased rabbits at different infection doses, suggesting a correlation with time of death post-infection. Histopathological analysis further revealed that an infection dose of 1 × 10^5^ TCID50 caused severe neural tissue damage. In the 90-day-old pig model, pigs challenged with the GDWS2 strain exhibited more severe respiratory and neurological symptoms than those infected with the JM strain, with a final mortality rate of 33% (2/6). Viral kinetics monitoring showed that nasal, oral, and anal swab viral loads in the GDWS2 group remained consistently higher than those in the JM group throughout the infection period. Histopathological analysis revealed significantly higher viral copy numbers in 11 tissues—including the spleen, lung, kidney, and central nervous system (cerebrum, cerebellum, pons, medulla oblongata)—in the GDWS2 group compared to the JM group. Additionally, characteristic lesions, such as pronounced neurotropism and perivascular cuffing, were observed in the central nervous system.

In summary, the PRV GDWS2 strain identified in this study represents a novel recombinant variant of genotype II. Moreover, *in vitro* and *in vivo* experiments have confirmed that its virulence surpasses that of the highly pathogenic JM variant strain isolated in 2021. GDWS2 induced more severe respiratory and neurological symptoms, along with a higher mortality rate, in 90-day-old pigs. Additionally, it exhibited enhanced neurotropism in tissues and an increased capacity for viral shedding.

## Data Availability

The original contributions presented in the study are included in the article/Supplementary material, further inquiries can be directed to the corresponding authors. The complete genome of the GDWS2 strain is deposited in the NCBI repository, with the accession number PV457541.

## Footnotes

^1^ https://wahis.oie.int/#/dashboards/country-or-disease-dashboard
