# Supplementary material for: Characteristics of the pseudorabies virus strain GDWS2 with severe neurological signs and high viral shedding capacity in pigs
Source: Front Vet Sci. 2025 Apr 14;12:1530765. doi: 10.3389/fvets.2025.1530765 (PMC12034708; doi:10.3389/fvets.2025.1530765)
Supplement: Supplementary file 1 [file Data_Sheet_1.docx]

Supplementary Material

# Supplementary Table

Table S1. The average score of pathological injury in various parts of the brain of pigs infected with PRV.

|  | Cerebrum | | Cerebellum | | Pons | | Bulbus rhachidicus | |
| --- | --- | --- | --- | --- | --- | --- | --- | --- |
|  | GDWS2 | JM | GDWS2 | JM | GDWS2 | JM | GDWS2 | JM |
| View 1 | adefgh | adefghi | bdegijk | deg | degh | dehi | eghij | adefh |
| View 2 | bcdeghi | aegh | deghi | dehjk | adefgh | degh | deghij | aij |
| View 3 | defhi | eh | deghi | ceh | adegh | adeghi | adefghi | ehij |
|  |  |  |  |  |  |  |  |  |
| View 1 | aefgh | deh | adeghi | adegh | aegj | dei | adefghij | deg |
| View 2 | degh | egh | adefghi | deg | adefh | degh | aeghi | adegi |
| View 3 | degh | egh | deghikl | eg | aegj | ae | adeghj | deh |
|  |  |  |  |  |  |  |  |  |
| View 1 | aegh | adegh | degh | cefghij | adefghi | dgj | deghij | egh |
| View 2 | degh | adefgh | abdegi | eh | adefghi | dgj | egij | egh |
| View 3 | efgh | adegh | adeghi | acdef | deghi | dgj | dej | aefgh |
|  |  |  |  |  |  |  |  |  |
| View 1 | acdefghi | degh | bcdefghijk | cefghij | adefghij | adfghi | acdfghij | degh |
| View 2 | abcdefghi | deghi | acdefghijk | ehik | deghi | adfghi | acefhij | degh |
| View 3 | abcdefghi | degh | cdefghik | efi | deghi | adfghi | cdefhij | degh |
|  |  |  |  |  |  |  |  |  |
| View 1 | defghi | deghi | adefg i | aehi | cdeghij | dehij | deghij | degh |
| View 2 | abdeghi | aefh | acdefghi | ehk | cdefgh | egh | adeghij | degh |
| View 3 | adefghi | adehi | cdefghk | efk | acdefghi | eghj | deghij | deghj |
|  |  |  |  |  |  |  |  |  |
| View 1 | abdefghi | egh | adefhi | dehjk | dghij | deghij | adeghi | degh |
| View 2 | bdegh | deh | abeghijk | cehij | adeghi | eh | adefghj | deghj |
| View 3 | deghi | aegh | degh | efi | deghij | deghij | adefgij | degh |
| Total score | 5.94^a^ | 4.17^b^ | 6.67^a^ | 4^b^ | 5.72^a^ | 4.22^b^ | 6.17^a^ | 4^b^ |
| *P*-Value | 0.045 | | 0.002 | | 0.034 | | 0.008 | |

(**A**) Inflammatory cell infiltration, (**B**) Eosinophilic inclusion body, (**C**) Hemorrhage, (**D**) Neurocyte lysis, (**E**) Vascular sheath, (**F**) Vascular dilation and congestion, (**G**) Gliosis, (**H**) Neurophagocytosis, (**I**) Neuronolysis, (**J**) Loose structure, (**K**) Unobvious protrusion of Purkinje cells, (**L**) Disappearance of Purkinje cells; Lowercase letters indicate P < 0.05.

# Supplementary Figures


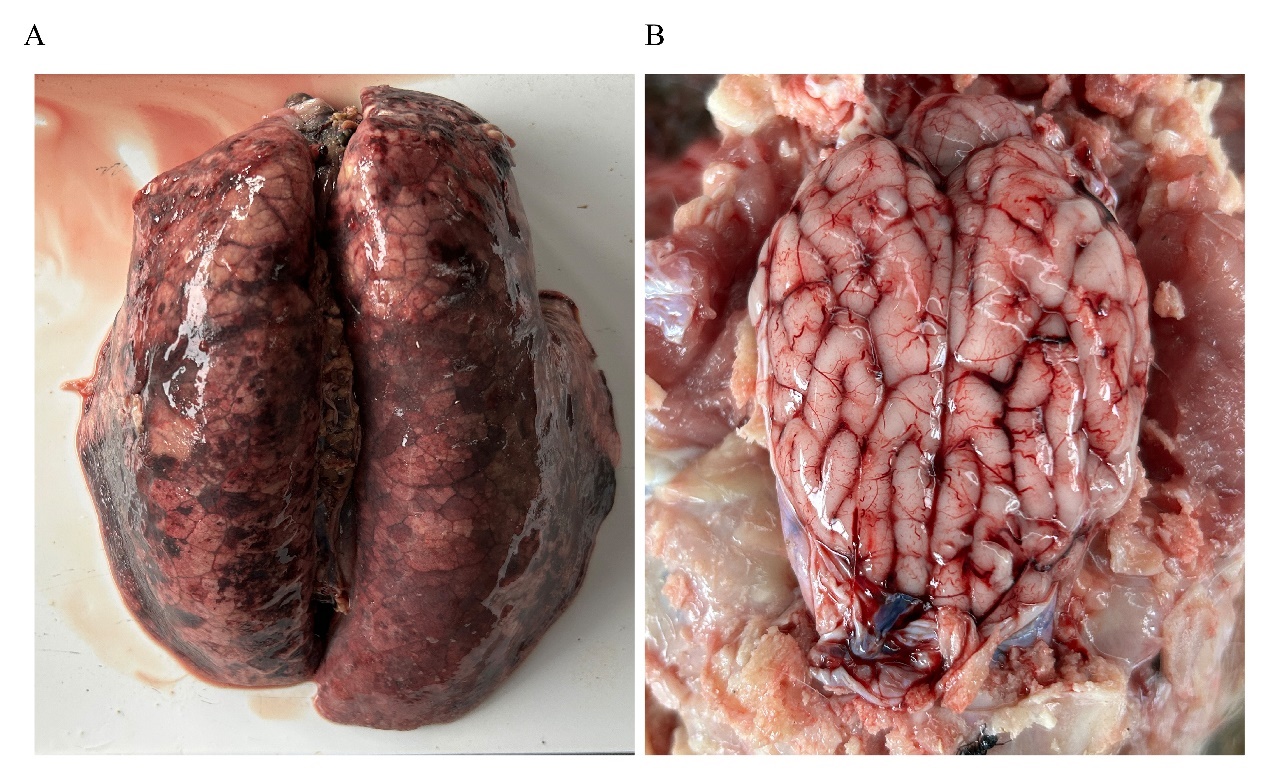


Fig. S1: Isolation and identification of pseudorabies virus GDWS2. (A) Lung damage of the pig where pseudorabies virus (PRV) GDWS2 was isolated. (B) Brain damage of the pig where PRV GDWS2 was isolated.


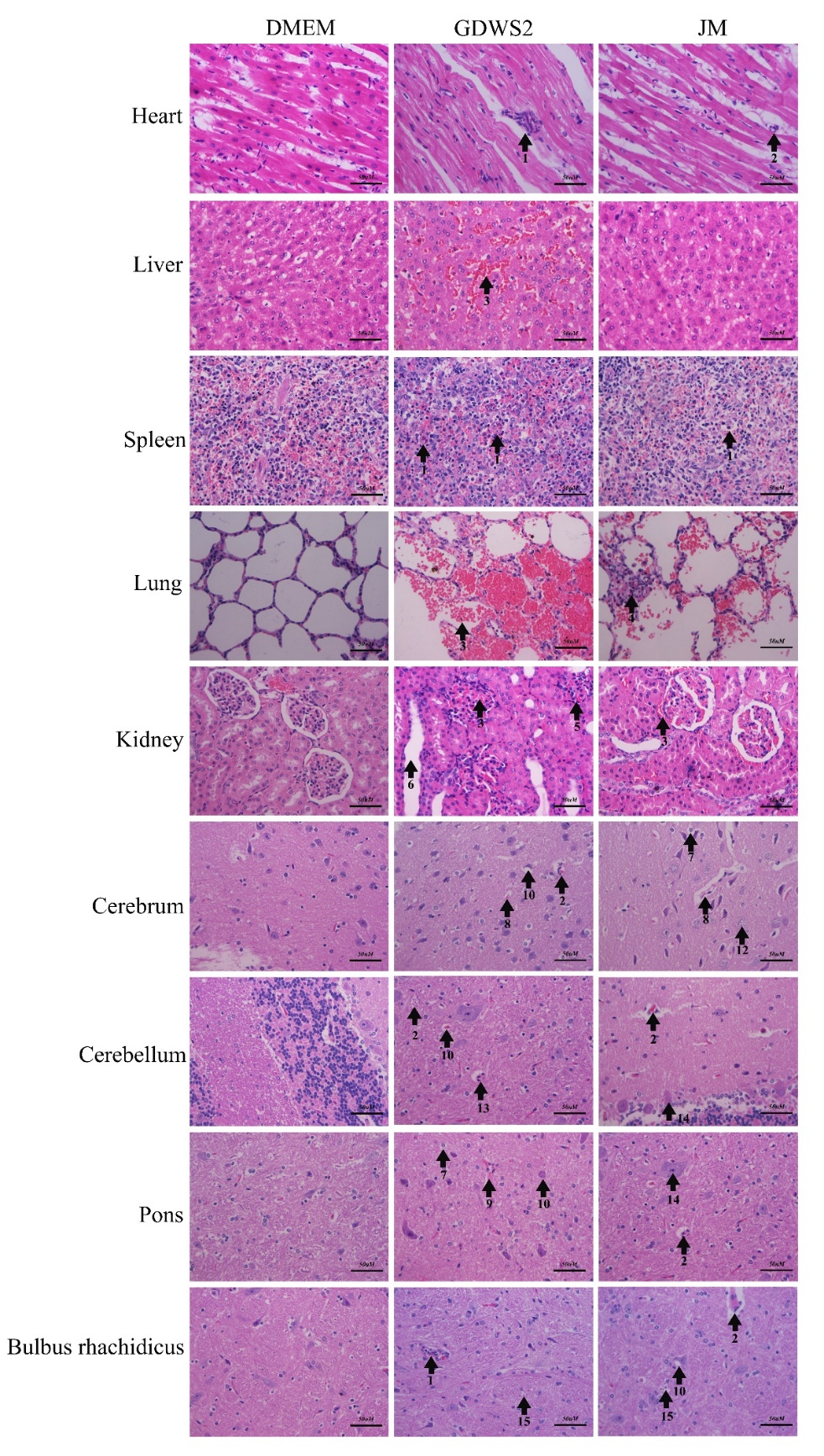


Fig. S2. Evaluation of viral virulence using rabbit models. All the pathological tissues were observed under a microscope (400× magnification). The arrow points to the lesion site. Tag: 1, Inflammatory cell infiltration; 2, Eosinophilic inclusion body; 3, Hemorrhage; 4, Alveolar swelling; 5, Glomerular swelling; 6, Renal tubular atrophy; 7, Neurocyte lysis; 8, Vascular sheath; 9, Vascular dilation and congestion; 10, Gliosis; 11, Neurophagocytosis; 12, Neuronolysis; 13, Loose structure; 14, Unobvious protrusion of Purkinje cells; 15, Disappearance of Purkinje cells.


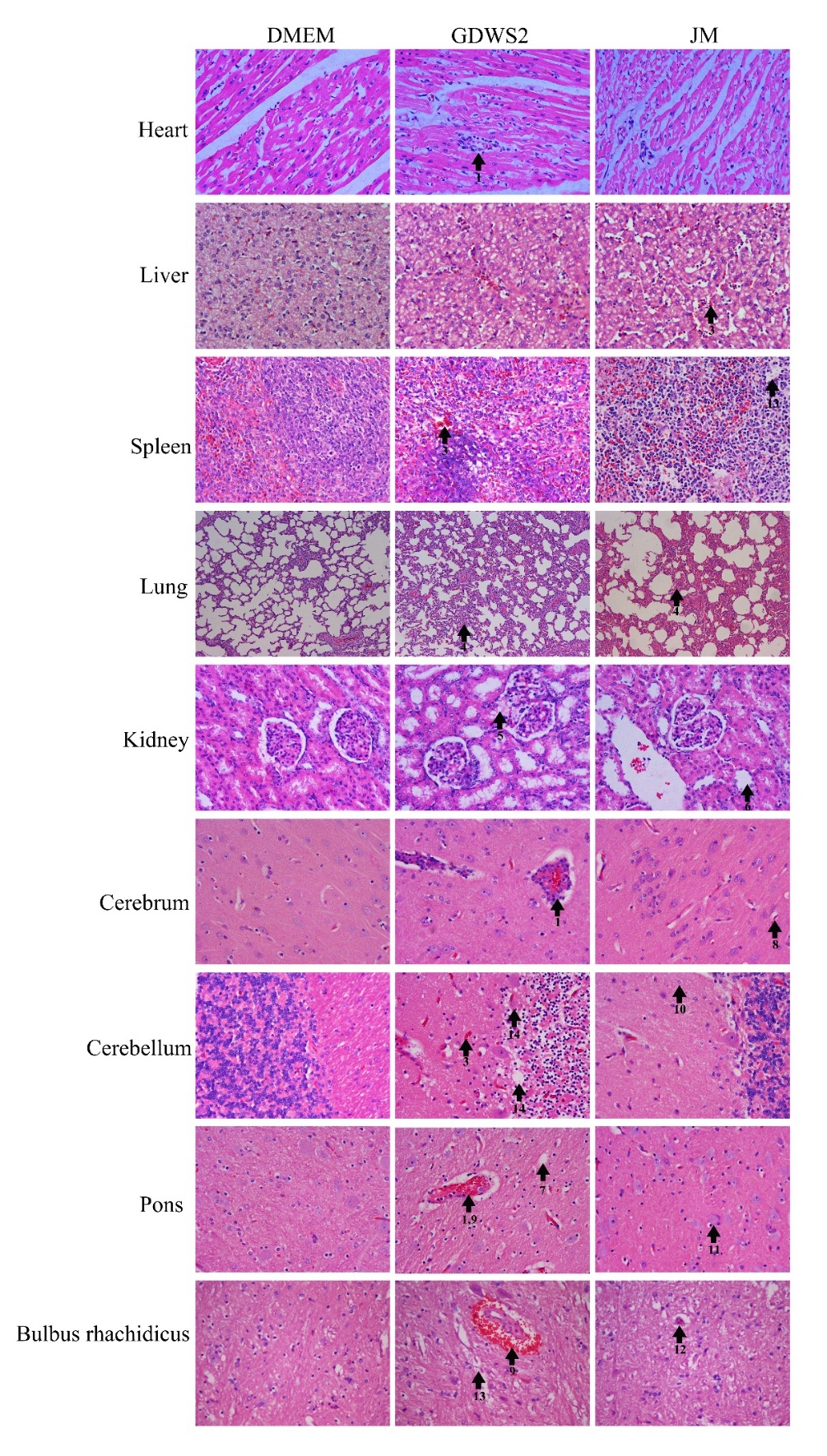


Fig. S3. Evaluation of viral virulence using pig models. All the pathological tissues were observed under a microscope (400× magnification). The arrow points to the lesion site. Tag: 1, Inflammatory cell infiltration; 2, Eosinophilic inclusion body; 3, Hemorrhage; 4, Alveolar swelling; 5, Glomerular swelling; 6, Renal tubular atrophy; 7, Neurocyte lysis; 8, Vascular sheath; 9, Vascular dilation and congestion; 10, Gliosis; 11, Neurophagocytosis; 12, Neuronolysis; 13, Loose structure; 14, Unobvious protrusion of Purkinje cells; 15, Disappearance of Purkinje cells.


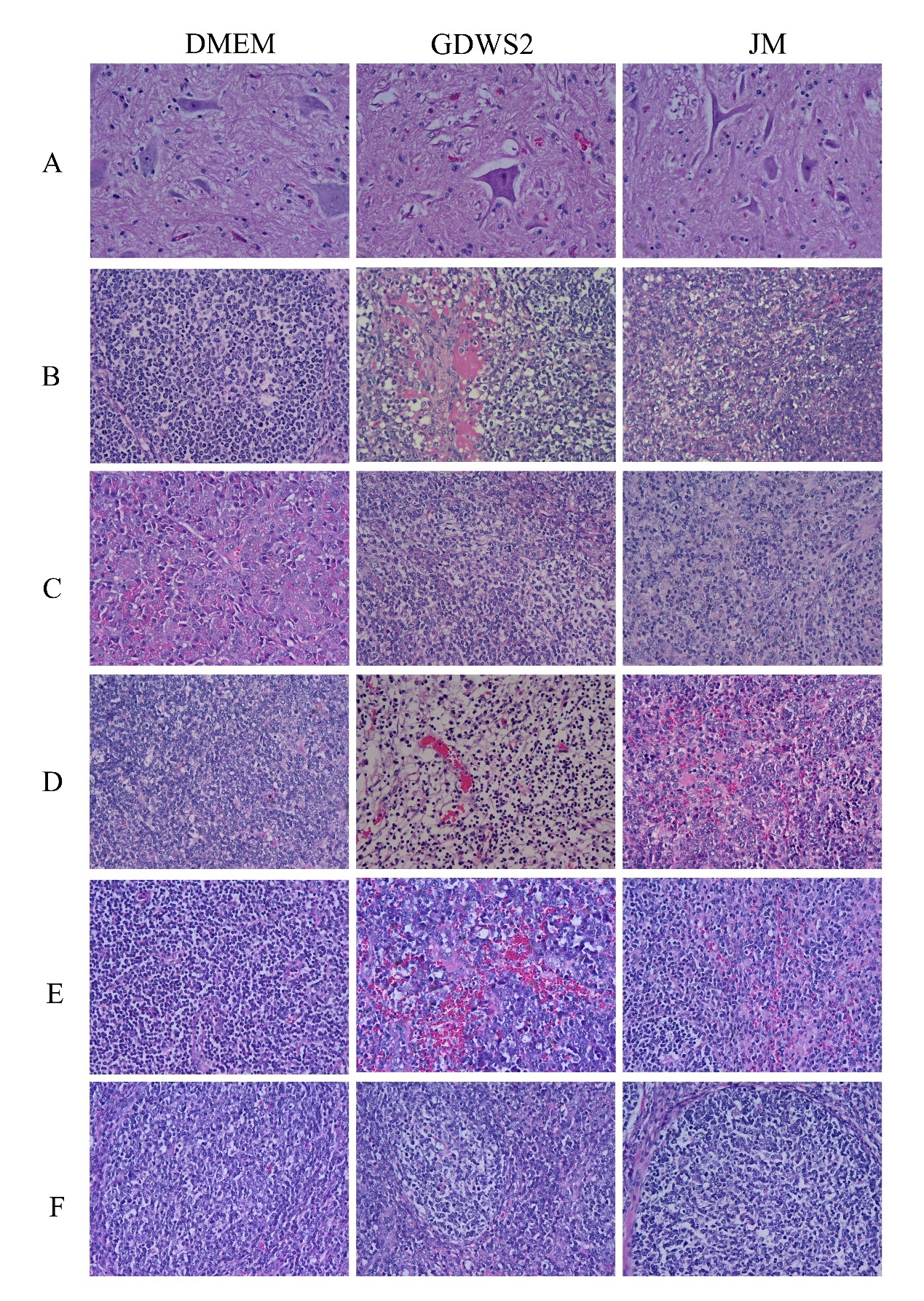


Fig. S4. Evaluation of viral virulence using pig models. (**A**) Medulla oblongata, (**B**) Hepatic hilar lymph nodes, (**C**) Splenic hilar lymph nodes, (**D**) Pulmonary hilar lymph nodes, (**E**) Submandibular lymph nodes, (**F**) Tonsil.
